# Detection of *Coxiella burnetii* in Ambient Air after a Large Q Fever Outbreak

**DOI:** 10.1371/journal.pone.0151281

**Published:** 2016-03-18

**Authors:** Myrna M. T. de Rooij, Floor Borlée, Lidwien A. M. Smit, Arnout de Bruin, Ingmar Janse, Dick J. J. Heederik, Inge M. Wouters

**Affiliations:** 1 Division of Environmental Epidemiology, Institute for Risk Assessment Sciences, Utrecht University, Utrecht, the Netherlands; 2 Centre for Infectious Disease Control (CIb), National Institute for Public Health and the Environment (RIVM), Bilthoven, the Netherlands; Institute for Health & the Environment, UNITED STATES

## Abstract

One of the largest Q fever outbreaks ever occurred in the Netherlands from 2007–2010, with 25 fatalities among 4,026 notified cases. Airborne dispersion of *Coxiella burnetii* was suspected but not studied extensively at the time. We investigated temporal and spatial variation of *Coxiella burnetii* in ambient air at residential locations in the most affected area in the Netherlands (the South-East), in the year immediately following the outbreak. One-week average ambient particulate matter < 10 μm samples were collected at eight locations from March till September 2011. Presence of *Coxiella burnetii* DNA was determined by quantitative polymerase chain reaction. Associations with various spatial and temporal characteristics were analyzed by mixed logistic regression. *Coxiella burnetii* DNA was detected in 56 out of 202 samples (28%). Airborne *Coxiella burnetii* presence showed a clear seasonal pattern coinciding with goat kidding. The spatial variation was significantly associated with number of goats on the nearest goat farm weighted by the distance to the farm (OR per IQR: 1.89, CI: 1.31–2.76). We conclude that in the year after a large Q fever outbreak, temporal variation of airborne *Coxiella burnetii* is suggestive to be associated with goat kidding, and spatial variation with distance to and size of goat farms. Aerosol measurements show to have potential for source identification and attribution of an airborne pathogen, which may also be applicable in early stages of an outbreak.

## Introduction

Q fever is a zoonotic disease caused by the bacterium *Coxiella burnetii*. In humans, Q fever can be acute or chronic and has a wide variety of clinical symptoms.[1] Ruminants, particularly goats and sheep, are the best known reservoir of *C*. *burnetii* and shedding occurs primarily during abortion or delivery.[2,3] Worldwide, Q fever is predominantly known as an occupational disease of veterinarians, farmers and abattoir workers.[4] However, several residential outbreaks have been described.[5–7] Between 2007 and 2010, the Netherlands experienced large residential outbreaks with a substantial disease burden in the general population paralleled by 25 notified fatal cases.[8] In total 4026 human cases were notified during this epidemic which peaked in 2009.[9] Severe control measures like mass culling of dairy goats and vaccination campaigns were implemented from December 2009 onwards. In the Netherlands, a relation between the human outbreak and living in the proximity of a goat farms was observed, in particular farms that experienced clinical signs of *C*. *burnetii* infection.[10] To a lesser extent also sheep farms were identified to be associated with human Q fever cases.[11]

Transmission of *C*. *burnetti* is considered to occur primarily through air. These outbreaks raised awareness for the importance of environmental airborne *C*. *burnetii* exposure. Spread of airborne *C*. *burnetii* likely occurs via aerosolized dust particles emitted by farms.[12,13] The *C*. *burnetii* spore-like form is very resistant to adverse conditions such as desiccation, can survive in soil and has a long survival time.[4,14] Geospatial modeling on *C*. *burnetii* transmission showed a potential role of environmental characteristics, and in particular, meteorological conditions.[15–17] However, these models have not been validated using exposure measurements. In addition, exposure measurements are important for validation of transmission models, assessment of exposure, strain identification by using molecular typing techniques, and to increase understanding of transmission routes. To our knowledge, only four studies[12,13,18,19] measured *C*. *burnetii* in air, of which only one in the outdoor air beyond farm premises. De Bruin et al were able to detect DNA of *C*. *burnetii* in aerosol samples in the vicinity of goat farms. However, the size of the particles sampled in this study is not well-defined and likely does not only reflect the particles that can enter the respiratory system. In addition, sampling was based on extremely short sampling times, hence a considerable sampling variability can be expected. So far, well designed larger scale exposure studies for C. *burnetii*, which focus on size-selective collection of particles that may be inhaled, are lacking.

As a first step to obtain information about *C*. *burnetii* exposure levels in residential areas, we conducted an extensive measurement campaign investigating the presence of *C*. *burnetii* in the outdoor air repeatedly at residences near goat farms. This study aimed to gain insight in spatial and temporal variation of *C*. *burnetii* by applying general approaches for assessment of environmental dust exposure.[20]

## Material and Methods

### Study design

**Fig 1:**
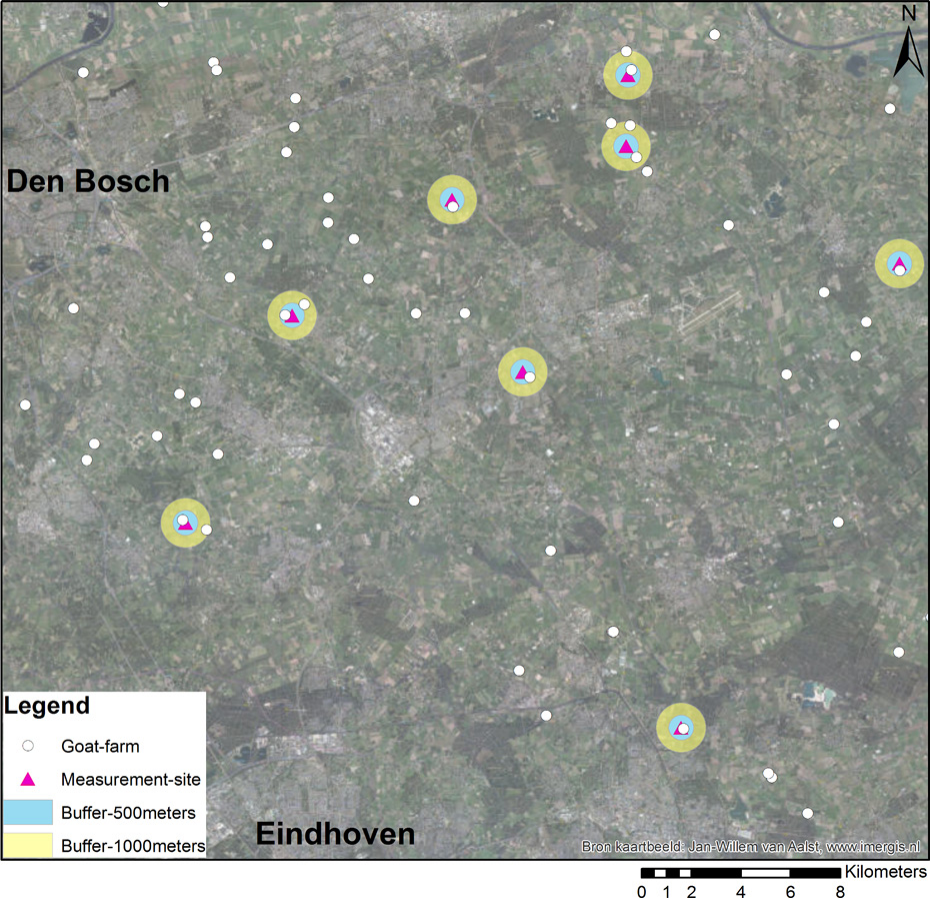
Overview of the geographical positions of measurement sites and goat farms in the research area situated in the province Noord-Brabant in the Netherlands.

Measurements were performed at eight measurement sites located in the east of Noord-Brabant, the province in the Netherlands where most Q fever cases had occurred. Sites were distributed over an area of 840 km^2^, which is characterized by a high goat farm density (Fig 1). Measurement installations were placed in residential gardens. Particulate Matter with a 50% cut-point aerodynamic diameter of 10 micrometer and less (PM10) were collected, representative for the size range of particles capable of entering the respiratory system. At each measurement location, a one-week average PM10 sample was collected for each week from March till September 2011. Thus all sites were sampled simultaneously over a total of 28 consecutive weeks. Some measurements were missing because of equipment failures and at some sites the measurements series were started later. As a result, the number of samples per location ranged from 24 to 26. Meteorological data was obtained from the local weather station at Volkel from the Royal Netherlands Meteorological Institute, situated in the measurement area.

### Sampling site selection

Coordinates of the location of all livestock farms and the number of licensed animals per farm in the year 2009, were obtained from the provincial database of mandatory environmental licenses for keeping livestock, as provided by the province of in Noord-Brabant. After the study had been conducted, licensed numbers of goats were reconfirmed with the provincial database for keeping livestock in 2012. In the Netherlands, bulk tank milk screening every two weeks has been mandatory for dairy goat and dairy sheep farms with at least 50 goats/sheep since 2009.[11] Bulk milk screening was commissioned by the government to be performed by the Food and Consumer Product Safety Authority in close collaboration with the Central Veterinary Institute and the Animal Health Service. As we did not have permission to sample at the farms, we used the bulk tank milk status from the national survey as a proxy for *C*. *burnetii* infection status. The status of each farm was obtained through the public overview of tank-milk positive farms published by ‘the Netherlands Food and Consumer Product Safety Authority’ accessed on January 24^th^ 2011.[21] Areas eligible for measurements were selected based on the list of registered goat farms. Sites had to meet the following criteria; (1) presence of residences close to a goat farm, (2) no other goat farm in a radius of 650 meter, and (3) covering a range in characteristics of the nearby goat farm (number of goats, bulk tank milk status).

Residents in eligible areas were informed about the study and were asked to participate. Field inspection of the premises of those willing to participate took place to ensure the garden was suited for the measurement installation. Participants granted permission for the placement of the air measurement installation in their garden.

### Spatial and temporal characteristics of sampling sites

Spatial characteristics possibly related to airborne *C*. *burnetii* were computed by means of geographical software (ArcGIS, version 10.2.2). The number of goats, sheep and cattle in buffer zones of respectively 500 and 1000 meters around the measurement sites were determined. Furthermore the distance and angle between the measurement site and the nearby goat farm was calculated. Air pollution is known to dilute with increasing distance from the source. To take into account this dilution effect;, a variable, distance weighted number of goats on the nearby farm, was defined by dividing the number of goats on the nearby farm by the squared distance to the farm. One-week average meteorological conditions were determined for temperature, relative humidity, precipitation, wind speed, wind direction, and duration of sunshine. The percentage of time that the measurement site was downwind from the nearby farm during the sampling week was calculated based on the hourly wind direction and the angle between the measurement site and the farm centered in a 40 degree range. Information on farm specific temporal kidding data, obtained from the ‘Netherlands Enterprise Agency’ (RVO) was only available for 4 measurements sites.

Hermans et al previously published on the kidding season course over the year based upon numbers of born goats/sheep on 117 farms (all goat farms, except four sheep farms), showing a similar yearly kidding pattern for the years 2006–2010.[22] We thus estimated monthly kidding percentages—number of goats born per calendar month divided by total number of born goats in the year—in 2010 and regarded those to be representative for the common kidding season pattern of 2011.

### Sampling method

Airborne particulate matter with a median aerodynamic diameter of 10 μm (PM10) was sampled at 2.5 m height with Harvard Impactors (Air Diagnostics and Engineering Inc., Naples, ME, USA) on 37-mm Teflon filters with 2 μm pore size (Teflo W/ring; Pall Corporation, Michigan, Ann Arbor, USA). Derenda pumps (LVS 3.1; Comde-Derenda GmbH, Stahnsdorf, Germany) were used to maintain the flow of 10 l/min. To avoid filter overloading, only half of each hour air was collected during 7 consecutive days so that effectively a 79-h sample was collected per week. Field blank control samples were collected, consisting of a clean filter put into a Harvard Impactor attached to the measurement pole during the one week sampling period, but not connected to the pump. A field blank control was collected every sampling week, and each week this was performed at a different site. In addition, a limited number of duplicate measurements was carried out by taking side by side measurements at the same location. All samples were stored within 24 hours after collection at -20°C.

### Sample processing

Particle mass was established by gravimetric analysis using an analytical microbalance with precision of 1 μg. Filters were conditioned for 24 hours prior to pre- and post-weighing in a weighing room with a controlled temperature (21±0.5°C) and relative humidity (35±5%) as described previously.[20] Subsequently, filters were processed in a sequential extraction procedure. Filters were transferred to 50-ml tubes (Greiner Bio-one) containing 5 ml of pyrogen-free water (Aqua B. Braun) supplemented with 0.05% Tween 20 (Calbiochem, United States). After shaking for 1 hour in an end-over-end roller, tubes were centrifuged at 1000xg for 15 minutes and 1 ml of supernatant was stored for endotoxin analysis (not covered in this paper). To the tube with the filter and the remaining 4 ml of fluid, 100 μl lysostaphin (1 mg/ml; Sigma Aldrich, USA), and 20 μl lysozyme (50 mg/ml; Sigma Aldrich, USA) was added and incubated for 35 minutes at 37°C. Next, 400 μl protease K (20 mg/ml; Roche Diagnostics Nederland B.V, Almere, the Netherlands) was added and incubated for 10 minutes at 55°C. Samples were heated for 10 minutes at 95°C. After adding 36 ml NucliSens Lysisbuffer (Biomerieux-diagnostics, Marcy l’Etoile, France) the tubes were homogenized for 1 hour on an end-over-end roller. To each tube, 50 μl *Bacillus thuringiensis* spore suspension (dilution 1:10; Raven Labs, Omaha, USA) was added to serve as an internal control for both DNA extraction and qPCR amplification. NucliSens Magnetic Bead DNA extraction was performed according to the manufacturer’s protocol (Biomerieux-diagnostics, Marcy l’Etoile, France). Small modifications at the end of the procedure were made to enhance the elution process, as described before.[23] The final concentrated DNA extract had a volume of 50 μl and samples were stored at -20°C.

### Coxiella burnetti qPCR

*Coxiella burnetii* DNA presence was determined by qPCR on a Lightcycler 480 Instrument (Roche Diagnostics Nederland B.V, Almere, the Netherlands). Characterization of qPCR performance was guided by the MIQE guidelines (minimum information for publication of qPCR experiments) and is previously described by de Bruin et al (2011).[23] The assay is highly efficient as only 10.6 or 10.4 gene copies per reaction are required for the targets com1 and IS*1111* respectively, and has high reproducibility.[23]Briefly, the qPCR assay detected three targets, two for *C*. *burnetii* (*com1* and *IS1111*) and one for *Bacillus thuringien*sis internal control target (*cry1b*). This assay showed a high comparability with other assays at different testing centers within an interlaboratory comparison of C. burnetii real-time PCR assays.[24] The control target validates both for DNA extraction and PCR amplification. Characterization of qPCR performance of the *C*. *burnetii* PCR has been described previously.[23] Samples were tested in triplicate using 3 μl of DNA extract per reaction well. A sample was scored positive for *C*. *burnetii* if the internal control target was positive and at least one of the two *C*. *burnetii* targets showed a positive signal in at least one of the triplicates.

### Statistical analysis

Statistical analyses were carried out using SPSS for Windows (version 22) and R studio for Windows (version 3.0.2). Differences in percentage of *C*. *burnetii* positive samples between locations were tested by a Chi-square test, followed by post-hoc binomial tests to identify which location differed significantly from the overall proportion positives. To check for a temporal trend, binary logistic regression was performed to analyze the association between a positive outcome and measurement week. Associations between spatial agricultural and temporal meteorological variables with *C*. *burnetii* positivity were analyzed by means of univariable generalized linear mixed logistic regression with random intercept per measurement site. Variables with a cut-off value of p<0.20 were selected for multivariable generalized linear mixed logistic regression with random intercept per measurement site. The final model was obtained with Backward Likelihood Ratio (LR) selection with a cut-off value of p<0.05. Univariable logistic regression on number of goats born per week was performed on the subset of sites for which farm specific kidding data was available. Kidding data was log-transformed because of the skewed distribution.

## Results

*Coxiella burnetii* DNA was detected in 56 samples (28%) out of the 202 qPCR analyzed field samples. Due to measurement failures, qPCR outcomes of 16 filters are lacking, and are not taken into account in the analyses. The *C*. *burnetii* DNA levels were low, as usually only the multi-copy *C*. *burnetii* target was detected at levels just above the limit of detection and well below the limit of quantification. In total, 29 field blank controls were collected of which 2 showed positive results for *C*. *burnetii* DNA.

**Fig 2:**
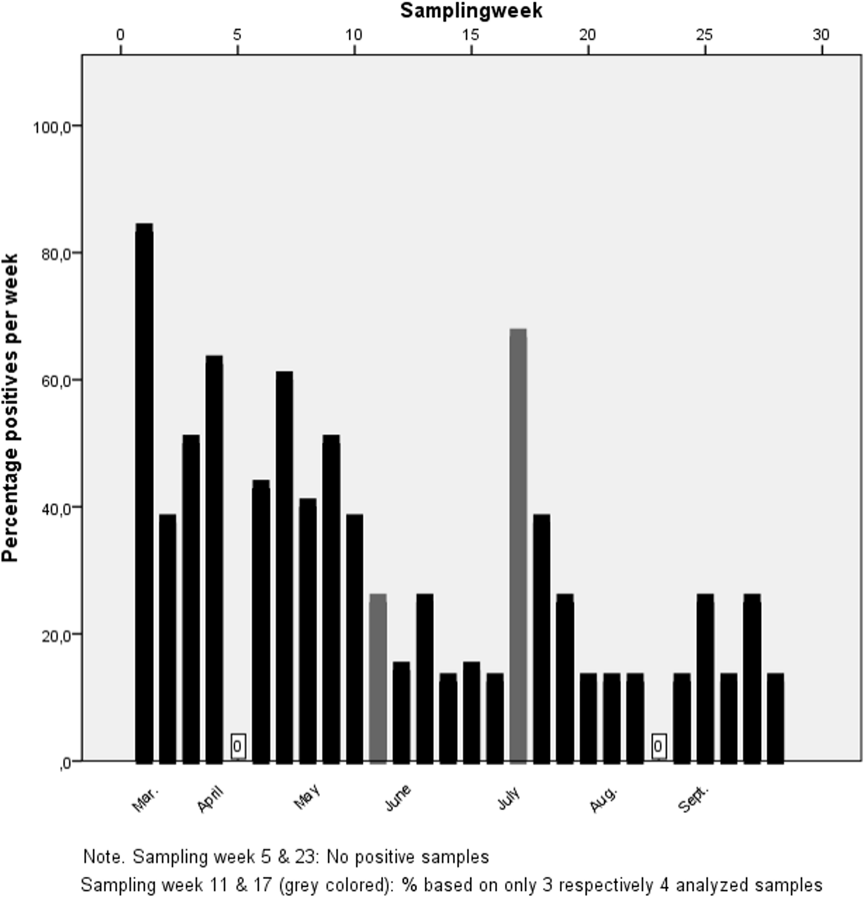
Percentage of positive samples for *C*. *burnetii* DNA per week from March–September 2011.

Table 1 provides an overview of the location characteristics and the number of positive samples, samples in which C. *burnetii* DNA was detected, per location. Percentages of positive samples differed significantly between locations (χ^**2**^(7) = 14.762, p<0.05), with location 5 having a significantly higher proportion of positives. Overall sampling was conducted during a 28 week time period, the percentage of positive samples differed considerably over the sampling weeks ranging from 0% to 83.3%. Samples were more often positive at the start of the sampling campaign, in spring, than later in the year; see Fig 2. (OR = 0.92, 95%CI: 0.88–0.96, unit is 1 week)

**Table 1:** Descriptive characteristics and percentage of PM10 samples tested positive for *C*. *burnetii* DNA by sampling location.

Site	Percentage of PM10 samples tested positive for C. *burnetii* (%)	Number of PM10 samples analyzed per location (N)	Distance to nearest goat farm (m)	Number of goats on nearest goat farm^1^ (N)	Nearest goat farm tested bulk milk positive ^2^
1	16.0%	25	253	300	No
2	20.0%	25	349	1325	No
3	19.2%	26	271	2480	No
4	28.0%	25	144	725	Yes
5	56.0%	25	91	688	Yes
6	26.9%	26	249	1400	No
7	30.8%	26	256	1500	Yes
8	25.0%	24	591	2266	No

^1^ Number of goats is based on numbers of goats allowed according to the environmental license.

^2^ Bulk milk positive outcomes for *C*. *burnetii* listed on January 24^th^ 2011.

Spatial, temporal and spatial-temporal characteristics were analyzed, see Table 2 for descriptive statistics. Univariable logistic regression with temporal and spatial characteristics indicated a potential link associated with a higher odds of a *C*. *burnetii* positive sample with lower temperatures, lower humidity levels, and less precipitation. Regarding the spatial variables, positive bulk-tank milk status of the farm was found to be a statistically significant determinant of *C*. *burnetii* positivity. Furthermore, the ‘squared distance weighted number of goats’ variable was associated statistically significantly with *C*. *burnetii* outcome, but not the separate variables: number of goats on farm and distance to farm (Table 3).

**Table 2:** Descriptive statistics of the spatial, temporal and spatial-temporal characteristics.

Variable type	Variables	AM*	Med*	SD*	Min*	Max*
Spatial	Number of goats^1^ on nearest goat farm (in hundreds)	13.48	14.00	7.18	3.00	24.80
	Distance^2^ to goat farm (per 100 m)	2.77	2.56	1.42	0.91	5.91
	Number of goats (in hundreds)/ squared distance (per 100 m)	2.75	2.28	2.38	0.47	8.27
	Number of goats within 500m buffer (per hundred)	10.57	13.25	7.46	0.00	24.80
	Number of goats within 1000m buffer (per hundred)	25.63	14.00	29.03	3.00	95.61
	Number of sheep within 500m buffer (per hundred)^3^	0.00	0.00	0.00	0.00	0.00
	Number of sheep within 1000m buffer (per hundred)	1.34	0.45	2.58	0.00	8.02
	Number of cows within 500m buffer (per hundred)	6.67	6.12	4.84	0.62	13.30
	Number of cows within 1000m buffer (per hundred)	18.00	14.54	11.68	2.73	37.77
Temporal	Temperature (per °C)	14.71	15.14	3.01	5.77	18.21
	Relative Humidity (per %)	74.45	77.56	9.27	57.48	86.51
	Precipitation (per 10 mm)	13.78	10.03	13.01	0.00	51.46
	Wind speed (per 0.1 m/s)	33.27	32.04	9.63	16.60	49.25
	Sunshine (per 0.1 hours)	2.58	2.28	1.02	1.29	4.82
Spatial-temporal	Natural logarithm of number of goats born on nearest) goat farm per measurement week^4^	1.06	.69	1.26	0.00	4.04
	Percentage of time sampling site downwind from the nearest goat farm	10.19	7.20	9.42	0.00	48.40

* AM = Arithmetic Mean; Med = Median; SD = Standard Deviation; Min = Minimum value in range; Max = Maximum value in range.

^1^ Number of goats on farm represents the number of goats (in hundreds) on the nearest farm from the measurement site.

^2^ Distance is the distance between measurement site and nearest goat farm measured in hundreds of meters.

^3^ There were no sheep within a 500 m buffer at each measurement site

^4^ Farm specific kidding data present for 4 out of 8 measurement sites

**Table 3:** Univariable associations between the presence of *C*. *burnetii* in PM10 and spatial, temporal and spatial-temporal characteristics.

Variable type	Variables	OR	95% CI	P-value
Spatial	Number of goats^1^ on nearest goat farm (in hundreds)	0.98	0.92–1.04	0.418
	Distance^2^ to nearest goat farm (per 100 m)	0.80	0.61–1.08	0.105
	Number of goats (in hundreds)/ squared distance (per 100 m)	1.23	1.08–1.40	0.002
	Number of goats within 500m buffer (per hundred)	0.99	0.93–1.05	0.606
	Number of goats within 1000m buffer (per hundred)	1.00	0.98–1.01	0.576
	Number of sheep within 500m buffer (per hundred)^3^	-	-	-
	Number of sheep within 1000m buffer (per hundred)	0.98	0.82–1.17	0.817
	Number of cows within 500m buffer (per hundred)	0.98	0.90–1.08	0.659
	Number of cows within 1000m buffer (per hundred)	0.99	0.95–1.02	0.372
	Nearest goat farm bulk-milk positive^4^	2.26	1.16–4.53	0.011
Temporal	Temperature (per °C)	0.82	0.73–0.91	<0.001
	Relative Humidity (per %)	0.96	0.93–0.99	0.016
	Precipitation (per 10 mm)	0.97	0.94–1.00	0.030
	Wind speed (per 0.1 m/s)	1.00	0.97–1.04	0.906
	Sunshine (per 0.1 hours)	1.41	1.04–1.93	0.028
	Estimated monthly percentage of number of goats born in the Netherlands (%)^5^	1.08	1.04–1.12	<0.001
Spatial-temporal	Natural logarithm of number of goats born on nearest farm per measurement week ^6^	1.30	0.93–1.84	0.134
	Percentage of time sampling site downwind from the nearest goat farm	1.01	0.97–1.04	0.717

^1^ Number of goats on farm represents the number of goats (in hundreds) on the nearest farm from the measurement site.

^2^ Distance is the distance between measurement site and nearest goat farm measured in hundreds of meters.

^3^ There were no sheep within a 500 m buffer at each measurement site

^4^ Bulk-milk positive outcomes for *C*. *burnetii* tested on January 24^th^ 2011

^5^ Monthly percentage of number of goats born in the Netherlands estimated on basis of the paper of Hermans et al. [[22]]

^6^ Subset analyses for farm specific kidding data present for 4 out of 8 measurement sites

**Fig 3:**
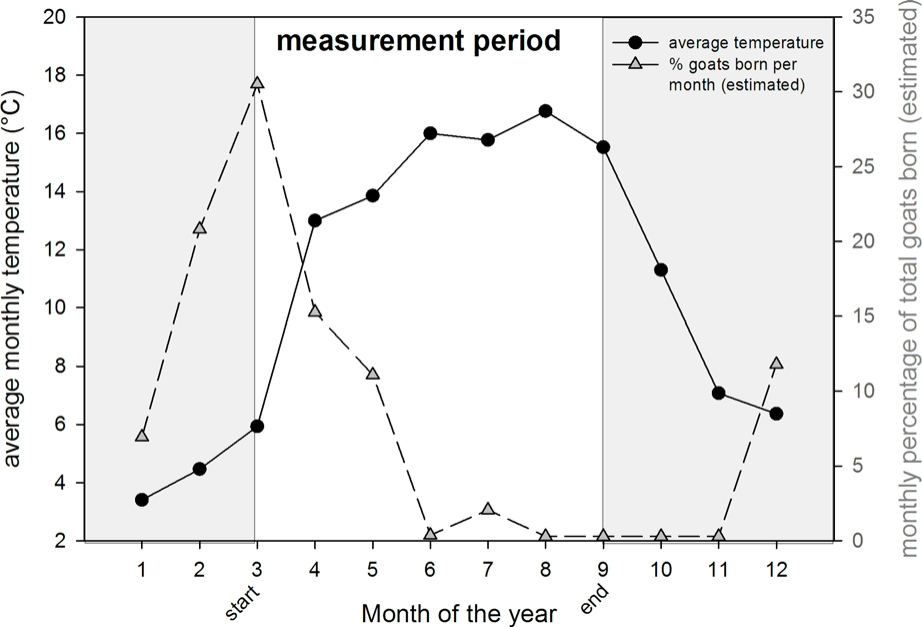
Overview of monthly averaged outdoor temperature and pattern of kidding season (estimated monthly percentages of goats born in the Netherlands based on the paper of Hermans et al [22]).

No significant association between farm-specific kidding data and *C*. *burnetii* positivity was found in the subset for which this data was available. In contrast, a strong significant association was found for the monthly percentage goats born in the Netherlands and number of positive samples (Table 3) The monthly kidding numbers were highly negatively correlated with monthly averaged temperatures (Pearson correlation of -0.973, p<0.001;, Fig 3). Duration of sunshine and humidity were highly correlated with each other (Pearson correlation of -0.714, p<0.001), which precluded the combined use of these variables in a multiple regression analysis.

A final multivariable model (R^2^ = .204 (Nagelkerke); model χ^2^(3) = 30.71, p<0.001) showed temperature, humidity and distance weighted number of goats; to be independently associated with *C*. *burnetii* positivity (Table 4). With increasing temperature, or humidity, the odds of a positive *C*. *burnetii* sample is lower. With increasing goat numbers and/or decreasing distance between the measurement site and the goat farm, the higher the odds of a positive sample.

**Table 4:** Significant temporal and spatial associations to the presence of *C*. *burnetii* in PM10, mutually adjusted in the multivariable model.

Variable	OR per unit	25%–75% percentiles	OR per IQR*	95% CI OR per IQR	P-value
Temperature (°C)	0.82	13.83–16.99	0.52	0.37–0.73	<0.001
Humidity (%)	0.96	71.07–83.29	0.60	0.39–0.94	0.024
Number of goats/squared distance	1.26	0.65–3.39	1.89	1.31–2.76	<0.001

Note. Number of goats on farm represents the number of goats (in hundreds) on the nearest farm from the measurement site. Squared distance is the distance squared between measurement site and nearest goat farm measured in hundreds of meters.

* Inter Quartile Range (IQR) is 3.16 for temperature, 12.22 for humidity and 2.74 for distance weighted number of goats

## Discussion

We were able to detect *C*. *burnetii* DNA in ambient air, collected at sites in residential areas near goat farms after a major Q-fever outbreak which affected thousands of individuals. In general, observed *C*. *burnetii* DNA levels were very low, often just above the detection limit and levels did not exceed the quantification bound. Nonetheless, spatial as well as temporal characteristics were identified to be associated with presence of *C*. *burnetii* in PM10 dust samples. This has not been achieved before by other studies concerning airborne measurements of *C*. *burnetii*. Distance weighted number of goats on the nearby farm and temporal characteristics were independently associated with presence of *C*. *burnetii* in PM10.

This is the first large regional scale study in which spatial and temporal variation in *C*. *burnetii* could be linked with agricultural and meteorological characteristics. Our findings give more insight into factors of influence for airborne transmission and yield insights which can be relevant for the proper management of Q fever during future outbreaks. These results also indicate that molecular analysis of environmental samples might be of use for the identification of sources and could contribute to source attribution in the early stages of Q-fever outbreaks when detailed epidemiological information is not yet available.

### Low DNA levels

Samples positive for *C*. *burnetii* displayed Cq values that were higher than those needed for proper quantification by using a standard curve. The number of *Coxiella* bacteria present in the samples were therefore probably very low. Part of this is due to the fact that measurements were performed after the peak of the Q fever outbreak, which ceased after implementation of drastic intervention measures. Culling of goats at *C*. *burnetii* positive farms started end of 2009 and continued until mid-2010. Vaccination of goats against *C*. *burnetii*, became mandatory in 2009, which has been shown to reduce bacterial shedding considerably.[25] Consequently, reduction of bacterial load in the environment could be expected. This was reflected in reduction in human Q fever incidence in The Netherlands, of which the peak was in 2009 with 2354 new cases, 504 cases were reported in 2010, and 81 in 2011.[26] Ambient air measurements by De Bruin et al[13] indicated a reduction in *C*. *burnetii* DNA content between 2010 and 2009. As the number of cases dropped even more in 2011, this presumably is also true for the level of exposure which may explain measured low levels in the current study. The earlier measurements likely yielded higher *C*. *burnetii* DNA levels, although direct comparisons are difficult because of the differences in sampling technique and particularly in sampling time.[13]

Because of the binominal nature of the *C*. *burnetii* data we simply analysed the data using logistic regression analysis in the current study. With larger measurement series one also might expect incidental higher values during peak exposure events. These should be evaluated with other techniques which can deal with zero inflated data, e.g. by using imputation or Tobit regression for left censored data, or beta-Poisson statistics, to obtain an unbiased average concentration estimate, as commonly applied for many infectious agents.[27]

Sampling variation was investigated by means of duplicate (parallel) measurements. These measurements were performed at 3 locations from June until September. Duplicate measurements showed a very high Pearson correlation of 0.936 (p<0.001) for particle mass. Detection of *C*. *burnetii* DNA showed a higher degree of variability. Out of 38 sampling pairs, 29 paired samples were both negative, and of the remaining 9 sampling pairs, only one sample in a pair was positive. No sample pair had a positive *C*. *burnetii* outcome in both samples simultaneously. This underlines the possibility of a naturally occurring high variability of *C*. *burnetii* DNA content in the air as *Coxiella* bacteria are probably not homogenously distributed in the air, in particular as a result of the low concentration observed. Because positive samples only contained very low DNA levels, stochastic processes have a relatively big effect on the possibility of detection We expect spatial variation to be high because of a limited amount of local sources. Therefore, after bacteria are shed by animals, which is not continuously, distribution in the ambient air depends on random eddies. As many particles are present in the air and air speeds can be high, a high dilution effect can be expected even in small areas. We speculate on the possibility of bacteria clumped together on bioaerosols, so making variability even higher. In contrast, particle mass is characterized by a high regional background and local sources are of minor importance so spatial variation is less resulting in highly correlated duplicate measurements.[28] Unexpectedly, two of the field blank samples were positive for *C*. *burnetii* DNA, resulting in a percentage positives amongst the blanks of 6.9%. Although no forced airflow passed these filters, the possibility of naturally occurring wind flow in the Harvard Impactor and deposition of *C*. *burnetii* DNA on the filter, cannot be excluded, and likely explains the positive field blank samples. Contamination during sample processing is thought to be unlikely as Coxiella bacteria are not part of human flora and do not belong to the common environmental species Cross-reaction of other DNA to the primers used is deemed improbable as specificity of the PCR was reported to be high.[23] Furthermore the control targets used to check for both the DNA extraction and PCR amplification, were negative. Either way, irrespective of what might have occurred, the effect on the sample outcomes is expected to be randomly distributed and will not affect the results of determinant analysis.

### Spatial and temporal variation

In spite of the difficulties related to airborne pathogen exposure measurements discussed above, we were able to identify an association with spatial as well as temporal characteristics. The variation spatially was associated with distance weighted number of goats surrounding the measurement site. These findings are indicative of a higher exposure when close to goat farms and with increasing numbers of goats. This is the first study to show such relationship for airborne exposure measurements. A spatial relation between the distance of the nearest (infected) farm and the residential locations of human cases was found as well in several epidemiological studies which attempted to identify the source of the Q fever outbreak by exploring the correlation between occurrence density and distance.[10,29] An ecological study using reported Q fever epidemic data showed for total numbers of goats and goat densities a clear association with human case status.[15] Similar findings were reported by Smit et al using electronic medical record data from approximately 90000 individual from general practitioners in the region.[30] Our study shows that identification of risk factors can also be based on airborne exposure measurements, with the advantage of independency of a sufficient number of human cases unlike epidemiology based methods. Thus in case of future outbreaks, air sampling can be implemented at an early stage to potentially accelerate source identification.

Univariable analysis showed that a positive bulk tank milk status of the goat farm was related to higher odds of detecting *C*. *burnetii* DNA in a PM10 sample, but this association did not hold in the multiple regression model. A considerable number of positive air samples was found at sites nearby farms negative for bulk tank milk. This suggests that shedding is not only restricted to bulk tank milk positive farms. Bulk tank milk tests were designed for detecting within-herd prevalence of at least 15 percent.[31] This implies that *C*. *burnetii* positive animals could have been present on bulk tank negative farms.[31] In addition, the relation between shedding in the milk and shedding by other means is not clearly understood. Bulk tank milk testing is probably not the best method to assess the presence of *C*. *burnetii* bacteria on a farm. However it is the method used in the national surveillance system in the Netherlands and restrictive measures are enforced for farms on the basis of these results.

A clear and statistically significant association was identified between sampling week and the number of positive samples; the percentage of positive samples per week decreased with increasing week number. This temporal variation can be caused by differences over time in *C*. *burnetii* load at the source, but also other time-related factors like meteorological conditions, may explain this time related pattern. The effect of kidding season could not be assessed independent from other temporal variables, because of a strong correlation with temperature. The influence of the kidding season on human cases has been shown before and can be expected to be present in this time series as well.[32,33] The level of airborne *C*. *burnetii* is expected to be high as shedding of *C*. *burnetii* to the environment is highest during parturition/abortion.[34] Increase in odds of a positive sample was observed in univariable analysis with increasing monthly percentages of goats born in the Netherlands. However, this effect was not observed when using data from 4 measurement sites where individual farm kidding data was available for the farm closest to the measurement site. The quality of this data obtained by farmers self-registration could not be validated, furthermore kidding on farms further away might also have an impact but this could not be verified. The monthly kidding pattern was not taken into account in multiple regression analyses not only because of (multi)collinearity problems but also because numbers were estimated and available only per month instead of per week.

Meteorological factors like relative humidity, wind velocity, temperature, precipitation and atmospheric stability are known to affect transport of airborne bacteria.[35] Dry and windy weather are known to favor the transmission of dust particles itself as well as re-suspension of particles into the air, these are thus likely advantageous conditions for the spreading of *C*. *burnetii*. Indeed we found that a lower relative humidity is associated with increased *C*. *burnetii* positivity. In contrast, we found a negative association between an increase in temperature and *C*. *burnetii* positivity. Part of this might be due to (multi)collinearity problems with the course of the kidding season, as during spring when temperatures are lower, kidding takes place.

Although a considerable number of spatial and temporal characteristics were taken into account; preliminary research indicates that data on manure, operational kidding and abortion numbers and vegetation might be of potential interest to explain spatial and temporal variation as well.[16,22,34,36,37]

### Health implications

Only low levels of *C*. *burnetii* DNA were found at the different sites. However when viable and inhaled, these can pose a considerable health risk as dose-response relations demonstrate high infectivity in humans.[38] Brooke et al estimated the probability of a single viable *C*. *burnetii* causing infection or illness in humans to be 0.44 and 0.12 respectively. [38] The impact of aerosolization on the viability of *Coxiella* bacteria is unclear, however some certainly survive given that humans have been infected by airborne exposure.[11] Since only DNA was analyzed in the air samples, the viability of the bacteria that are present in our aerosol samples is unknown, which has to be realized when making inferences about implications for human health. Thus far, no studies have reported cultivation of *C*. *burnetii* from air samples, and this seems impracticable due to the risks and difficulties associated with *C*. *burnetii* cultivation. Nevertheless, it would be of interest to have insight in the ratio of dead versus viable *C*. *burnetii* bacteria in the air, which would aid interpreting the DNA levels observed. With knowledge on viability, assessments of residential exposures to living *C*. *burnetii* bacteria can be made.

### Implications future outbreaks

This study shows the potential of airborne measurements to contribute to source identification and source attribution of a pathogen. Retrospectively, PM10 sampling in combination with molecular analysis of *C*. *burnetii*, in areas with both dense livestock and residential areas or even a high human population density could have contributed to identification of risk factors in the early stages of the Q fever epidemic in the Netherlands. The main disadvantage of source identification by means of epidemiological data is the need for sufficient human cases in order to obtain accurate conclusions. However, given pathogen levels vary over time and over space, a structured approach is required including long-term sampling at a sizable number of sites with a well-defined spatial distribution. Because the setup of a profound measurement network takes time, which in the case of an epidemic is limited, a continuous monitoring network should be considered. Sites should be strategically located in a livestock dense area. If levels of pathogens are measured continuously, sudden changes can be picked up immediately, enabling identification of sources at an early stage.Additionally, when an outbreak amongst animals with a zoonotic agent is detected, more agent-specific defined sampling campaigns should be implemented. Environmental sampling inside the farm and at farm premises can give valuable insights with respect to modes of transmission, which can support in deciding which intervention measures are relevant to prevent or stop an outbreak.[39] This would aid defining the environmental and public health impact of such an outbreak.

To conclude, spread of *C*. *burnetii* by air is driven by multiple complex processes and several diverse factors are expected to play a role. More insight was gained in temporal and spatial variability in exposure levels, despite the difficulties inherent in airborne measurement studies of pathogenic bacteria. The measurement series clearly show a seasonal pattern of *C*. *burnetii* DNA levels in the air. Furthermore our results suggest a combined influence of proximity of the goat farm and number of goats on that farm. This study shows the potential contribution of airborne measurements to source identification and attribution of a pathogen.
